# Cerebrospinal fluid in tuberculous meningitis exhibits only the L-enantiomer of lactic acid

**DOI:** 10.1186/s12879-016-1597-9

**Published:** 2016-06-07

**Authors:** Shayne Mason, Carolus J. Reinecke, Willem Kulik, Arno van Cruchten, Regan Solomons, A. Marceline Tutu van Furth

**Affiliations:** Centre for Human Metabolomics, Faculty of Natural Sciences, North-West University (Potchefstroom Campus), Private Bag X6001, Potchefstroom, 2531 South Africa; Laboratory Genetic Metabolic Diseases, Department of Clinical Chemistry, Academic Medical Center, University of Amsterdam, Amsterdam, The Netherlands; Department of Paediatrics and Child Health, Faculty of Medicine and Health Sciences, Stellenbosch University, PO Box 19063, Tygerberg, 7505 South Africa; Department of Paediatric Infectious Diseases–Immunology and Rheumatology, Vrije Universiteit Medical Centre, De Boelelaan 1117, 1081HV Amsterdam, The Netherlands

**Keywords:** Cerebrospinal fluid (CSF), Enantiomers, L- and D-lactic acid, Tuberculous meningitis (TBM), Ultra-performance liquid chromatography–electrospray ionization–tandem mass spectrometry (UPLC–ESI–MS/MS)

## Abstract

**Background:**

The defining feature of the cerebrospinal fluid (CSF) collected from infants and children with tuberculous meningitis (TBM), derived from an earlier untargeted nuclear magnetic resonance (NMR) metabolomics study, was highly elevated lactic acid. Undetermined was the contribution from host response (L-lactic acid) or of microbial origin (D-lactic acid), which was set out to be determined in this study.

**Methods:**

In this follow-up study, we used targeted ultra-performance liquid chromatography–electrospray ionization–tandem mass spectrometry (UPLC–ESI–MS/MS) to determine the ratio of the L and D enantiomers of lactic acid in these CSF samples.

**Results:**

Here we report for the first time that the lactic acid observed in the CSF of confirmed TBM cases was in the L-form and solely a response from the host to the infection, with no contribution from any bacteria. The significance of elevated lactic acid in TBM appears to be that it is a crucial energy substrate, used preferentially over glucose by microglia, and exhibits neuroprotective capabilities.

**Conclusion:**

These results provide experimental evidence to support our conceptual astrocyte–microglia lactate shuttle model formulated from our previous NMR-based metabolomics study — highlighting the fact that lactic acid plays an important role in neuroinflammatory diseases such as TBM. Furthermore, this study reinforces our belief that the determination of enantiomers of metabolites corresponding to infectious diseases is of critical importance in substantiating the clinical significance of disease markers.

**Electronic supplementary material:**

The online version of this article (doi:10.1186/s12879-016-1597-9) contains supplementary material, which is available to authorized users.

## Background

Lactic acid is a common metabolite and occurs as two, optically active stereoisomers (enantiomers) — L-lactic acid and D-lactic acid.

In humans, the L enantiomer is considered the normal, physiological form of lactic acid. The endogenous D form is also found in humans, but in nanomolar amounts due to methylglyoxal metabolism [1], which converts acetone derivatives to glutathione. The D enantiomer typically originates from gut microbiota, such that it can be detected in the blood and urine up to the millimolar range. Elevation of D-lactic acid typically occurs as a result of gut trauma or gastrointestinal disorder, for example, in jejuno-ileal bypass for obesity [2], appendicitis [3, 4], inflammatory bowel syndrome [5], and even in patients with chronic fatigue syndrome [6] and type 2 diabetes [5]. The main source of elevated D-lactic acid in urine, however, is in patients with short bowel syndrome [7].

It is known that the gut possesses its own unique microbiotic environment; it is also generally believed that the brain is a fairly sterile environment without its own microbiota, with the microglia in the circulating cerebrospinal fluid (CSF) actively eliminating any microbial incursions beyond the blood–brain barrier (BBB). However, in pathological conditions where an invading pathogen successfully transgresses the BBB and an infection occurs in the central nervous system (CNS), any subsequent metabolic perturbations may be difficult to trace and interpret. In the case of bacterial meningitis, in which bacteria infect the meninges, with disastrous consequences due to neuroinflammation, there have been numerous reports of highly acidotic CSF owing to the presence of lactic acid [8–10]. A unique form of bacterial meningitis, caused by *Mycobacterium tuberculosis* (Mtb), is tuberculous meningitis (TBM). What makes this form of meningitis unique, and dangerous, is that it has a slow, insidious onset which often leads to accumulated damage due to neuroinflammation, resulting in irreparable neural damage, or even death. It is a disease that is difficult to diagnose in its early stages and fatal if recognized at a late stage. Of the various CSF diagnostic markers for TBM, lactic acid is one that has been reported in a limited number of studies [11–13]. In a previous study we examined a cohort of infants and children with TBM by means of an untargeted ^1^H nuclear magnetic resonance (NMR)-based metabolomics approach [14]. The most noticeable feature that we detected was highly elevated lactic acid (7.36 ± 2.36 mM) in the CSF of these cases compared to the normal, age-related reference range (1.65 ± 0.63 mM) [15]. The presence of highly elevated lactic acid in CSF is not unique to meningitis cases: in patients with cerebral malaria there was significantly higher CSF lactic acid recorded (9.0 ± 5.3 mM [16]; 6.0 ± 1.0 mM [17]) in those who died compared to survivors. Yao et al. [18] reported that CSF lactic acid levels reflect the severity of metabolic impairment of the brain in patients with hepatic encephalopathy. Thus, knowledge of increased CSF lactic acid levels in response to neuropathology is not new; however, distinguishing the respective roles of the L and D forms has not been attempted hitherto.

In our NMR-based metabolomics study [14] the high levels of lactic acid, together with several other statistically significant metabolites, led to the formulation of the hypothesis: ‘The host’s response to neural infection results in an “astrocyte–microglia lactic acid shuttle” (AMLS) that operates in neuroinflammatory diseases, such as TBM’; thereafter, a conceptual model describing the AMLS was constructed. We are therefore seeing that lactic acid, previously considered an unimportant by-product of CNS metabolism, is now receiving more attention as it appears to play an essential role in normal neural homeostasis — in the astrocyte–neuron lactic acid shuttle (ANLS) [19] — and possibly has an important role in neuropathological states. For example, lactic acid is neuroprotective in cerebral ischemia [20], a condition often associated with TBM.

In the present investigation, we used the CSF samples collected in our previous study and employed a derivatization method adapted from Scheijen et al. [5] to differentiate the enantiomers of lactic acid present. We analysed the samples using the highly sensitive, targeted method of ultra-performance liquid chromatography–electrospray ionization–tandem mass spectrometry (UPLC–ESI–MS/MS). We report here for the first time that highly lactic acidotic CSF from infants and children with confirmed TBM exhibits only the L-enantiomer — hence it is a response solely by the host to the infection — and then discuss the relevance of the phenomenon of elevated lactic acid in this example of a neuroinflammatory disease.

## Methods

### Sampling

The experimental group consisted of infants and children (<13 years of age) (*n* = 20) from the Western Cape region of South Africa, who were directed from local clinics to the paediatric unit of Tygerberg Hospital, Cape Town, on suspicion of meningitis, based on clinical symptoms. Upon admission to hospital, a lumbar CSF sample was taken for differential diagnosis, a portion of which was stored at −80 °C for this study, which was used to confirm a diagnosis of TBM. A diagnosis of TBM was based on the procedure of the uniform research case definition of Marais et al. [21], practiced in our clinical setting. Only children with ‘definite’ and ‘probable’ TBM were included in the present patient group. TBM was classified as ‘definite’ when CSF demonstrated acid-fast bacilli on microscopy, a positive Mtb culture and/or passed a positive CSF Mtb commercial nucleic acid amplification test in a child with symptoms or signs suggestive of the disease. TBM was classified as ‘probable’ according to a scoring system based on clinical, CSF and neuroimaging criteria, as well as evidence of extraneural TB. Clinical details of the patients are described in the Supplementary Information of Mason et al. [14]. For the purpose of the present study the stage of TBM, as well as the glucose concentration in 16 out of the 20 CSF samples, were made available, as shown in Fig. 2. We also collected a urine sample from our subjects upon admission to hospital. Urine is often the biofluid of choice for investigating the various potential sources of lactic acid, as well as providing for a non-invasive mode of sample collection. A limitation in using urine remains the unpredictable fluctuation in the concentration of targeted metabolites linked to the disease state of the patients. Written consent was obtained from the caregiver and assent if the child was older than 7 years and competent to do so. This study was approved by the Human Research Ethics Committee of Stellenbosch University, South Africa (study no. N11/01/006) — informed consent and assent forms are given as additional information in Additional file 1.

### Chemicals

The chemicals used as standards were: sodium L-lactic acid ≥99.0 % (Sigma-Aldrich 71718, CAS: 867-56-1); sodium D-lactic acid ≥99.0 % (Sigma-Aldrich 71716, CAS: 920-49-0); sodium L-lactic acid-3,3,3-d3 (CDN isotopes D-2646, CAS: 79-33-4).

The following chemicals were used in sample preparation and analyses: (+)-O,O’-Diacetyl-L-tartaric anhydride (DATAN) (Sigma-Aldrich 358924, CAS: 6283-74-5); acetonitrile HPLC supragrade (Biosolve 01203502, CAS: 75-05-8); MilliQ water (Millipore, CAS: 7732-18-5); acetic acid 100 % (Merck 1.00063.1000, CAS: 64-19-7); ammonium formate (Sigma-Aldrich 25204, CAS: 540-69-2); dichloromethane (Lab-Scan AR1040A, CAS: 75-09-2); perchloric acid (Sigma-Aldrich 244252, CAS: 7601-90-3); formic acid 98–100 % (Merck 1.00264.1000, CAS: 64-18-6).

### Sample preparation and UPLC-ESI-MS/MS analysis

A dilution series of L- and D-lactic acid and a 2.5 mM L-lactic acid-d_3_ internal standard (IS) solution were prepared in advance for the calibration curve. A fresh diacetyl-L-tartaric anhydride (DATAN) derivatization solution was prepared by dissolving 250 mg of DATAN in 4 mL dichloromethane and 1 mL acetic acid. Samples were prepared by combining 50 μL CSF / 100 μL urine with 20 μL internal standard solution and 300 μL acetonitrile (ACN) in an Eppendorf tube. Samples were vortexed and centrifuged for 10 min at 3000 RPM in order to remove proteins and other macromolecules. The supernatant was transferred to a clean glass vial and dried under nitrogen gas at 40 °C. To this, 100 μL DATAN solution was added, vortexed and incubated at 75 °C for 30 min. Once again the solution was dried under nitrogen gas at 40 °C and then reconstituted in 200 μL ACN / H_2_O (1/2 v / v) for UPLC-ESI-MS/MS analysis. For the UPLC: eluent A (2.5 mM ammonium formate, pH = 3.6) and eluent B (100 % acetonitrile) were used. Samples were analysed on a Waters Acquity UPLC hyphenated to a Quattro premier XE triple quadrupole mass spectrometer using negative ion electrospray ionization — details given in Additional file 1.

After derivatization with DATAN, L- and D-lactic acid were separated from each other on the UPLC column and measured by MS/MS in MRM mode. Use of solvent delay time avoided the introduction of salts in the MS. Results were quantified in terms of the stable isotope-labelled analogue of L-lactic acid. The peak areas corresponding to component-specific transitions were converted via calibration lines to concentrations.

## Results

**Fig. 1:**
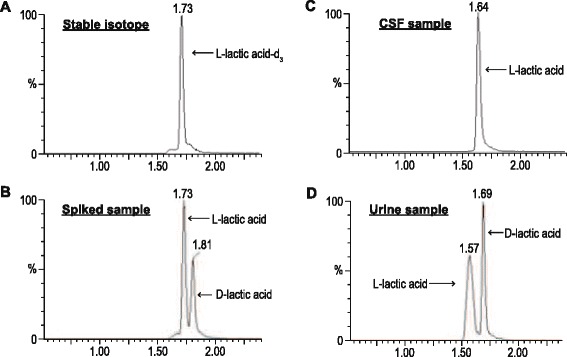
Representative chromatograms depicting **a** definitive identification of L-lactic acid using the stable isotope (L-lactic acid-d_3_); **b** clear differentiation of L and D forms of lactic acid in the spiked sample; **c** in CSF, complete lack of D form of lactic acid with only the L form present; and **d** presence of both L and D forms of lactic acid in urine

Using a spiked sample containing both the L and D forms of lactic acid, and comparing the chromatogram qualitatively with the stable isotope (L-lactic acid-d_3_), it was evident that the UPLC–ESI–MS/MS method used in this study was able to differentiate between the two enantiomers (Fig. 1a and b). We were therefore able to identify and quantify the lactic acid content of the CSF samples from the 20 TBM cases, as described in the ‘Methods’ section. These results revealed that the lactic acid was exclusively in the L-form — no D-isomer was detected (Fig. 1c) — with a mean concentration of 5.2 mM (SD = 2.6; range: 1.5–10.5 mM).

We also compared the total lactic acid concentrations in the corresponding samples with those derived from the UPLC–ESI–MS/MS method used in the current study and with those from the NMR method of Mason et al. [14], and found a strong linear relationship between the two sets of results (*r* = 0.93), with a statistically significant positive correlation (0.86) and a statistically strong validation of the fit with an R^2^ value of 0.73. These data therefore confirm that both methods are highly comparable, demonstrating high reproducibility.

**Fig. 2:**
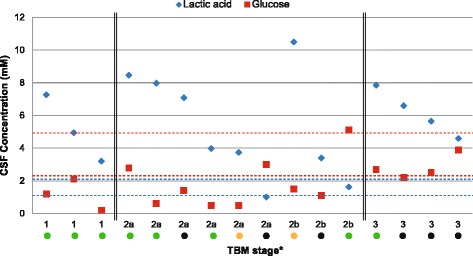
Scatterplot showing the concentration of lactic acid in the CSF samples and the corresponding CSF glucose (values not reported in text) over different stages of TBM disease (*dashed lines indicate respective reference ranges*). Outcome severity: *green circle* = normal; *yellow circle* = mild neurological problems (e.g., learning difficulties, visual impairment); *black circle* = severe neurological problems (e.g., partial paralysis, severe motor impairment, cranial nerve palsy). *TBM stage: 1 = Glasgow coma score (GCS) 15 and no focal neurology; 2a = GCS 15 plus focal neurology; 2b = GCS 11–14 with focal neurology; 3 = GCS <11

Of the 20 CSF samples examined, a clinician reported glucose values for 16 samples. Figure 2 is a scatterplot, showing both the lactic acid and glucose concentrations of the CSF, with the corresponding stage of the TBM disease. This figure illustrates the inverse relationship between lactic acid and glucose, together with the patient severity outcome after treatment. Additional statistics (shown in Additional file 1) support existing knowledge that glucose is significantly correlated with TBM stage and outcome severity. While L-lactic acid reached high concentrations in CSF samples in some patients from the present group (well above the reference ranges), the L-lactic acid did not correlate with statistical significance with the TBM stage or outcome severity — an observation that may, however, relate to the small sample size of the present group.

In addition to the CSF, the urine was examined of 7 patients upon admission to hospital. Both the L and D forms of lactic acid were detectable (illustrated in Fig. 1d), but in small amounts. The mean urine lactic acid was determined to be 98.4 μM for the L form (SD = 37.4; range: <50–155 μM) and 47.4 μM for the D form (SD = 56.7; range: nd–148 μM). The contribution of D-lactic acid from the gut microbiota cannot be distinguished from any possible influence of the Mtb, and the small amount of urinary lactic acid was well within the normal range. We conclude that urine is not a suitable biofluid for monitoring and assessing lactic acid for diagnostic purposes in TBM.

## Discussion

In the present investigation we identified and quantified the L and D enantiomers of lactic acid in the CSF of 20 cases of infants and children with confirmed TBM, using UPLC–ESI–MS/MS. This study was motivated by our NMR-based metabolomics study [14], in which the most prominent feature of the CSF of these TBM cases was highly elevated lactic acid. We therefore sought to determine the source and form of the lactic acid — was it a response from the host (which would generate the L-isomer), of microbial origin (which produces the D-isomer), or both? Because NMR is incapable of distinguishing the enantiomers of lactic acid, we conducted this follow-up study with the means to identify the separate isomers.

We have shown that both enantiomers were detectable in urine samples, but the elevated lactic acid in the CSF samples of the TBM patients consisted of only L-lactic acid, and hence was produced exclusively by the host with no contribution from the Mtb. Moreover, Fig. 2 shows that as the TBM disease progressed into later stages there was a drop in patient prognosis (i.e., increased outcome severity). Generally, in more advanced TBM stages, fewer energy substrates are available for microglia activity and decreased protection of neurons from the lactic acid. During this period, irreversible neurological damage can begin to occur. Lactic acid boosts energy production in the brain and increases neuroprotection, thus potentially improving the prognosis of patients, especially those at high risk. TBM patients in late stages of the disease who exhibit CSF with low lactic acid and glucose may be beyond help as the accumulated neural damage and lack of energy substrate can be fatal.

The origin and rationale of high levels of lactic acid produced by the host in response to a neuroinflammatory disease such as TBM is unclear. Interconversion of lactic acid and pyruvate occurs via lactate dehydrogenase, with increased lactic acid typically being associated with anaerobic respiration. Thus, elevated levels of lactic acid in the CSF due to neuroinflammation could be due to hypoxia caused by ischemia, or by increased glucose levels and hence increased flow through the glycolysis pathway. However, in TBM cases there are typically low levels of glucose in the CSF [22], corresponding to a CSF to serum glucose ratio less than 0.5 or an absolute CSF glucose concentration of less than 2.2 mM. Furthermore, several studies have shown no correlation between CSF lactic acid levels and cerebral blood flow (i.e., they are unrelated to ischemia) [23–25]. Thus, elevated lactic acid in CSF of TBM cases is unlikely to be due to anaerobic respiration but instead is possibly a product of temporarily increased flux in the glycolysis pathway.

The results reported here also provide experimental evidence which supports our proposed AMLS hypothesis [14]. The hypothesis postulates that, when the brain is in crisis due to neuroinflammatory-inducing infection, energy flow in brain metabolism is shifted away from the neurons and shunted towards the microglia. Hence, in neuroinflammatory infectious diseases, such as TBM, lactic acid produced by glycolysis in astrocytes participates in the activated immune response and, in association with ketones and gluconeogenic amino acids, is collectively directed from the neurons preferentially into microglia where it enters the mitochondrial citric acid cycle. This process contributes to oxidative phosphorylation and hence produces high levels of adenosine triphosphate (ATP) and forms of reactive oxygen species, such as hydrogen peroxide, required for degradation of the invading pathogen.

Further studies are needed to advance our understanding of the dynamics involved in this lactic acid phenomenon — for example: determining the rate at which microglia produce lactic acid under neuroinflammatory conditions such as TBM; an in vitro scenario testing whether a high level of lactic acid within the microglia reduces the uptake of interstitial lactic acid due to concentration gradients, accounting for the high concentrations of lactic acid in the CSF; more in-depth studies into transporters that shuttle lactate, namely, monocarboxylate transporters; and, in a clinical setting, determining the impact of adjunctive treatments involving direct infusion of sodium lactate into the CSF of advanced stage TBM patients.

While simple in design and execution, this study provides important information not reported before. The implications of these results are compelling in that the levels of lactic acid in the CSF, produced by the host, should be carefully considered by the clinician, especially regarding neuroinflammatory diseases. The reason for this is that lactic acid is used preferentially over glucose in such cases [26–30], provides a boost in neuroprotection [31, 32], and aids microglia energy demands in their bactericidal actions. Thus lactic acid has a potential beneficial role in the clinical management of neurological disorders [33].

## Conclusions

In summary, we have shown that the contribution from Mtb in the form of D-lactic acid in all our TBM-infected CSF cases was not demonstrable. The lactic acid consisted solely of the L-form, with the host being responsible for its high concentrations in the CSF. Beyond this study, the ramifications of determining the enantiomers — and by doing so the origin — of particular metabolites holds importance for future research. Knowledge of what is produced by the host and what is of microbial origin could revolutionize treatment regimes and allow us to understand disease pathogenesis and progress better. Therefore adaptation of the method presented should be made routine for common, optically active markers of disease to determine their respective enantiomers, particularly in the field of metabolomics.

## Abbreviations

ACN, acetonitrile; AMLS, astrocyte–microglia lactic acid shuttle; ANLS, astrocyte–neuron lactic acid shuttle; ATP, adenosine triphosphate; BBB, blood–brain barrier; CNS, central nervous system; CSF, cerebrospinal fluid; D-, dextrorotary; DATAN, diacetyl-L-tartaric anhydride; IS, internal standard; L-, levorotary; MRM, multiple reaction monitoring; Mtb, *Mycobacterium tuberculosis*; NMR, ^1^H nuclear magnetic resonance; SD, standard deviation; SI, supplementary information; TB, tuberculosis; TBM, tuberculous meningitis; UPLC–ESI–MS/MS, ultra-performance liquid chromatography–electrospray ionization–tandem mass spectrometry

## Additional file

Additional file 1: Cerebrospinal fluid in tuberculous meningitis exhibits only the L-enantiomer of lactic acid. (DOCX 148 kb)
