# Supplementary material for: Cerebrospinal fluid in tuberculous meningitis exhibits only the L-enantiomer of lactic acid
Source: BMC Infect Dis. 2016 Jun 7;16:251. doi: 10.1186/s12879-016-1597-9 (PMC4897924; doi:10.1186/s12879-016-1597-9)
Supplement: Additional file 1: — Cerebrospinal fluid in tuberculous meningitis exhibits only the L-enantiomer of lactic acid. (DOCX 148 kb) [file 12879_2016_1597_MOESM1_ESM.docx]

**Additional file 1**

**Cerebrospinal fluid in tuberculous meningitis exhibits only the L-enantiomer of lactic acid**

Shayne Mason^1^, Carolus J. Reinecke^1^, Willem Kulik^2^, Arno van Cruchten^2^, Regan Solomons^3^ and A. Marceline Tutu van Furth^4^

**Contents**

**S1 Detailed sample preparation and UPLC-ESI-MS/MS analysis** 2

Sampling 2

Chemicals 2

Sample preparation and UPLC-ESI-MS/MS analysis 3

**S2 Additional statistical analyses** 5

Parametric correlations 5

Nonparametric correlations 6

Regression 7

**S3 Raw data output** 8

**S4 Informed consent and assent forms** 9

**S1 Detailed sample preparation and UPLC-ESI-MS/MS analysis**

**Sampling**

The experimental group consisted of infants and children (<13 years of age) (n = 20) from the Western Cape region of South Africa, who were directed from local clinics to the paediatric unit of Tygerberg Hospital, Cape Town, on suspicion of meningitis, based on clinical symptoms. Upon admission to hospital, a lumbar CSF sample was taken for differential diagnosis, a portion of which was stored at –80°C for this study, which was used to confirm a diagnosis of TBM. A diagnosis of TBM was based on the uniform research case definition of Marais *et al.* [1]. Only children with ‘definite’ and ‘probable’ TBM were included. TBM was classified as ‘definite’ when CSF demonstrated acid-fast bacilli on microscopy, a positive Mtb culture and/or passed a positive CSF Mtb commercial nucleic acid amplification test in a child with symptoms or signs suggestive of the disease. TBM was classified as ‘probable’ according to a scoring system based on clinical, cerebrospinal fluid and neuroimaging criteria, as well as evidence of extraneural TB. Clinical details of the patients are described in the supplementary information of Mason *et al.* [2]*.* For the purpose of the present study the stage of TBM, as well as the glucose concentration in 16 out of the 20 CSF samples, were made available, as shown in Fig. 2. We also collected a urine sample from our subjects upon admission to hospital. Urine is often the biofluid of choice for investigating the various potential sources of lactic acid, as well as providing for a non-invasive mode of sample collection. A limitation in using urine remains the unpredictable fluctuation in the concentration of targeted metabolites linked to the disease state of the patients. Informed consent was obtained from all participants and this study was approved by the Human Research Ethics Committee of Stellenbosch University, South Africa (study no. N11/01/006).

**Chemicals**

The chemicals used as standards were: sodium L-lactic acid ≥99.0% (Sigma-Aldrich 71718, CAS: 867-56-1); sodium D-lactic acid ≥99.0% (Sigma-Aldrich 71716, CAS: 920-49-0); sodium L-lactic acid-3,3,3-d3 (CDN isotopes D-2646, CAS: 79-33-4).

The following chemicals were used in sample preparation and analyses: (+)-O,O’-Diacetyl-L-tartaric anhydride (DATAN) (Sigma-Aldrich 358924, CAS: 6283-74-5); acetonitrile HPLC supragrade (Biosolve 01203502, CAS: 75-05-8); MilliQ water (Millipore, CAS: 7732-18-5); acetic acid 100% (Merck 1.00063.1000, CAS: 64-19-7); ammonium formate (Sigma-Aldrich 25204, CAS: 540-69-2); dichloromethane (Lab-Scan AR1040A, CAS: 75-09-2); perchloric acid (Sigma-Aldrich 244252, CAS: 7601-90-3); formic acid 98-100% (Merck 1.00264.1000, CAS: 64-18-6).

**Sample preparation and UPLC-ESI-MS/MS analysis**

A dilution series of L- and D-lactic acid and a 2.5 mM L-lactic acid-d_3_ internal standard (IS) solution were prepared in advance for the calibration curve. A fresh diacetyl-L-tartaric anhydride (DATAN) derivatization solution was prepared by dissolving 250 mg of DATAN in 4 ml dichloromethane and 1 ml acetic acid. Samples were prepared by combining 50 μL CSF / 100 μL urine with 20 μL internal standard solution and 300 μL acetonitrile (ACN) in an Eppendorf tube. Samples were vortexed and centrifuged for 10 minutes at 3000 RPM in order to remove proteins and other macromolecules. The supernatant was transferred to a clean glass vial and dried under nitrogen gas at 40 °C. To this, 100 μL DATAN solution was added, vortexed and incubated at 75 °C for 30 minutes. Once again the solution was dried under nitrogen gas at 40 °C and then reconstituted in 200 μL ACN / H_2_O (1/2 v / v) for UPLC-ESI-MS/MS analysis. For the UPLC: eluent A (2.5 mM ammonium formate, pH = 3.6) and eluent B (100% acetonitrile) were used. Samples were analysed on a Waters Acquity UPLC hyphenated to a Quattro premier XE triple quadrupole mass spectrometer using negative ion electrospray ionization (ESI), and the following multiple reaction monitoring (MRM) parameters:

| Parent ion | Daughter ion | Dwell (s) | Cone (eV) | Collision (eV) | Delay (s) | Compound |
| --- | --- | --- | --- | --- | --- | --- |
| 305 | 89 | 0.200 | 10 | 8 | 0.005 | L- & D-lactic acid |
| 308 | 92 | 0.200 | 10 | 8 | 0.005 | L-lactic acid-d_3_ |

The UPLC column used was an Acquity UPLC bridged ethylene hybrid (BEH) C18 reverse phase with 1.7 µm particle size, 2.1 mm diameter and 100 mm length. Sample temperature was kept at 10 ºC in the autosampler and column temperature at 50 ºC. Injection volume was 10 µL. Source and desolvation temperatures were set at 150 ºC and 450 ºC, respectively. Capillary voltage was set at 1.50 kV. Cone and desolvation gas flow 50 L/h and 900 L/h, respectively. Sample run consisted of, first, blank + IS, followed by 5 calibration curve series samples, blank and then the prepared samples After derivatization with DATAN, L- and D-lactic acids were separated from each other on the UPLC column and measured by MS/MS in MRM mode. Use of solvent delay time avoided the introduction of salts in the MS. Results were quantified in terms of the stable isotope-labelled analogue of L-lactic acid. The peak areas corresponding to component-specific transitions were converted via calibration lines to concentrations.

**S2 Additional statistical analyses**

These statistics showed a significant correlation between Glasgow coma score and TBM stage at the 0.01 level. However, there was a significant correlation at the 0.05 level between Glasgow coma score and glucose, between TBM stage and poor prognosis, and between TBM stage and glucose. The significance of glucose is also seen in the regression model. Lactate did not show up as statistically significant in this small sample group.

| **Parametric correlations** |  |  |  |  |  |  |  |  |
| --- | --- | --- | --- | --- | --- | --- | --- | --- |
|  |  |  |  |  |  |  |  |  |
|  | | Glasgow coma score | Outcome severity score | TBM stage | lactate | glucose |  |  |
| Glasgow coma score | Pearson correlation | 1 | -.404 | -.862^**^ | .029 | -.526^*^ |  |  |
|  | Sig. (2-tailed) |  | .120 | .000 | .915 | .036 |  |  |
|  | N | 16 | 16 | 16 | 16 | 16 |  |  |
| Outcome severity score | Pearson correlation | -.404 | 1 | .514^*^ | -.122 | .215 |  |  |
|  | Sig. (2-tailed) | .120 |  | .041 | .654 | .425 |  |  |
|  | N | 16 | 16 | 16 | 16 | 16 |  |  |
| TBM stage | Pearson correlation | -.862^**^ | .514^*^ | 1 | .129 | .502^*^ |  |  |
|  | Sig. (2-tailed) | .000 | .041 |  | .634 | .047 |  |  |
|  | N | 16 | 16 | 16 | 16 | 16 |  |  |
| lactate | Pearson correlation | .029 | -.122 | .129 | 1 | -.213 |  |  |
|  | Sig. (2-tailed) | .915 | .654 | .634 |  | .428 |  |  |
|  | N | 16 | 16 | 16 | 16 | 16 |  |  |
| glucose | Pearson correlation | -.526^*^ | .215 | .502^*^ | -.213 | 1 |  |  |
|  | Sig. (2-tailed) | .036 | .425 | .047 | .428 |  |  |  |
|  | N | 16 | 16 | 16 | 16 | 16 |  |  |
| ** Correlation is significant at the 0.01 level (2-tailed). | | | | | | |  |  |
| * Correlation is significant at the 0.05 level (2-tailed). | | | | | | |  |  |

| **Nonparametric correlations** |  |  |  |  |  |  |  |
| --- | --- | --- | --- | --- | --- | --- | --- |
|  | | | Glasgow coma score | Outcome severity score | TBM Stage | lactate | glucose |
| Kendall's tau_b | Glasgow coma score | Correlation Coefficient | 1.000 | -.253 | -.770^**^ | .075 | -.331 |
|  |  | Sig. (2-tailed) |  | .240 | .000 | .704 | .096 |
|  |  | N | 16 | 16 | 16 | 16 | 16 |
|  | Outcome severity score | Correlation Coefficient | -.253 | 1.000 | .442^*^ | -.117 | .225 |
|  |  | Sig. (2-tailed) | .240 |  | .042 | .560 | .263 |
|  |  | N | 16 | 16 | 16 | 16 | 16 |
|  | TBM stage | Correlation Coefficient | -.770^**^ | .442^*^ | 1.000 | .085 | .409^*^ |
|  |  | Sig. (2-tailed) | .000 | .042 |  | .671 | .042 |
|  |  | N | 16 | 16 | 16 | 16 | 16 |
|  | lactate | Correlation Coefficient | .075 | -.117 | .085 | 1.000 | .025 |
|  |  | Sig. (2-tailed) | .704 | .560 | .671 |  | .892 |
|  |  | N | 16 | 16 | 16 | 16 | 16 |
|  | glucose | Correlation Coefficient | -.331 | .225 | .409^*^ | .025 | 1.000 |
|  |  | Sig. (2-tailed) | .096 | .263 | .042 | .892 |  |
|  |  | N | 16 | 16 | 16 | 16 | 16 |
| Spearman's rho | Glasgow coma score | Correlation Coefficient | 1.000 | -.292 | -.834^**^ | .089 | -.465 |
|  |  | Sig. (2-tailed) |  | .272 | .000 | .742 | .069 |
|  |  | N | 16 | 16 | 16 | 16 | 16 |
|  | Outcome severity score | Correlation Coefficient | -.292 | 1.000 | .500^*^ | -.162 | .268 |
|  |  | Sig. (2-tailed) | .272 |  | .048 | .548 | .316 |
|  |  | N | 16 | 16 | 16 | 16 | 16 |
|  | TBM stage | Correlation Coefficient | -.834^**^ | .500^*^ | 1.000 | .109 | .523^*^ |
|  |  | Sig. (2-tailed) | .000 | .048 |  | .689 | .038 |
|  |  | N | 16 | 16 | 16 | 16 | 16 |
|  | lactate | Correlation Coefficient | .089 | -.162 | .109 | 1.000 | .001 |
|  |  | Sig. (2-tailed) | .742 | .548 | .689 |  | .996 |
|  |  | N | 16 | 16 | 16 | 16 | 16 |
|  | glucose | Correlation Coefficient | -.465 | .268 | .523^*^ | .001 | 1.000 |
|  |  | Sig. (2-tailed) | .069 | .316 | .038 | .996 |  |
|  |  | N | 16 | 16 | 16 | 16 | 16 |
| ** Correlation is significant at the 0.01 level (2-tailed). | | | | | | | |
| * Correlation is significant at the 0.05 level (2-tailed). | | | | | | | |

| **Regression** |  |  |  |  |  |  |  |  |
| --- | --- | --- | --- | --- | --- | --- | --- | --- |
| Model Summary | | | | |  |  |  |  |
| Model | R | R square | Adjusted R square | Std. error of the estimate |  |  |  |  |
| 1 | .557^a^ | .311 | .205 | .9770 |  |  |  |  |
| a. Predictors: (Constant), glucose, lactate | | | | |  |  |  |  |
| ANOVA^a^ | | | | | | |  |  |
| Model | | Sum of squares | df | Mean square | F | Sig. |  |  |
| 1 | Regression | 5.591 | 2 | 2.796 | 2.929 | .089^b^ |  |  |
|  | Residual | 12.409 | 13 | .955 |  |  |  |  |
|  | Total | 18.000 | 15 |  |  |  |  |  |
| a. Dependent variable: TBM stage | | | | | | |  |  |
| b. Predictors: (Constant), glucose, lactate | | | | | | |  |  |
| Coefficients^a^ | | | | | | | | |
| Model | | Unstandardized coefficients | | Standardized coefficients | t | Sig. | 95.0% Confidence interval for B | |
|  |  | B | Std. error | Beta |  |  | Lower bound | Upper bound |
| 1 | (Constant) | 1.054 | .759 |  | 1.390 | .188 | -.585 | 2.694 |
|  | lactate | .103 | .098 | .247 | 1.049 | .313 | -.109 | .315 |
|  | glucose | .450 | .191 | .555 | 2.355 | .035 | .037 | .863 |
| a. Dependent variable: TBM stage | | | | | | | | |

**S3 Raw data ouput**

| **ID** |  | **L-lactic acid** |  | **D-lactic acid** |
| --- | --- | --- | --- | --- |
|  |  | (mmol/L) |  | (µmol/L) |
| CSF samples |  |  |  |  |
| CSF 138 |  | 7.3 |  | nd |
| CSF 139 |  | too little sample | |  |
| CSF 140 |  | 4.9 |  | nd |
| CSF 144 |  | 6.6 |  | nd |
| CSF 146 |  | 8.5 |  | nd |
| CSF 148 |  | 8.0 |  | nd |
| CSF 161 |  | 4.0 |  | nd |
| CSF 162 |  | 3.7 |  | nd |
| CSF 191 |  | 7.1 |  | nd |
| CSF 193 |  | 3.2 |  | nd |
| CSF 195 |  | 1.9 |  | nd |
| CSF 208 |  | 10.5 |  | nd |
| CSF 209 |  | 6.4 |  | nd |
| CSF 211 |  | 4.6 |  | nd |
| CSF 216 |  | 3.4 |  | nd |
| CSF 221 |  | 5.6 |  | nd |
| CSF 226 |  | 6.6 |  | nd |
| CSF 227 |  | 1.5 |  | nd |
| CSF 228 |  | 1.6 |  | nd |
| CSF 239 |  | 7.9 |  | nd |
| CSF 229 |  | 1.0 |  | nd |
| QC |  | 4.4 |  | nd |
|  |  |  |  |  |
|  |  |  |  |  |
|  |  | **L-lactic acid** |  | **D-lactic acid** |
|  |  | (µmol/L) |  | (µmol/L) |
| Urine samples |  |  |  |  |
| U 139 |  | <50 |  | nd |
| U 144 |  | <50 |  | nd |
| U 158 |  | 113 |  | 19 |
| U 162 |  | 92 |  | 16 |
| U 193 |  | 71 |  | 19 |
| U 201 |  | 61 |  | 35 |
| U 216 |  | 155 |  | 148 |

**S4 Informed consent and assent forms**

### PARTICIPANT INFORMATION LEAFLET AND CONSENT FORM

TITLE OF THE RESEARCH PROJECT:

Improving early diagnosis of tuberculous meningitis in children

**REFERENCE NUMBER: …..**

**PRINCIPAL INVESTIGATOR:** Dr RS Solomons

**ADDRESS: Ward G10, Tygerberg Children’s Hospital, Francie van Zijl avenue, Parow Valley**

**CONTACT NUMBER: xxxxxxxxxx**

You are being invited to take part in a research project. Please take some time to read the information presented here, which will explain the details of this project. Please ask the study staff or doctor any questions about any part of this project that you do not fully understand. It is very important that you are fully satisfied that you clearly understand what this research entails and how you could be involved. Also, your participation is **entirely voluntary** and you are free to decline to take part. If you say no, this will not affect you negatively in any way whatsoever. You are also free to withdraw from the study at any point, even if you do agree to take part.

This study has been approved by the Health Research Ethics Committee at Stellenbosch University and will be conducted according to the ethical guidelines and principles of the international Declaration of Helsinki, South African Guidelines for Good Clinical Practice and the Medical Research Council (MRC) Ethical Guidelines for Research.

## What is this research study all about?

- Your child has meningitis and is admitted in the Tygerberg Children’s Hospital.
- Meningitis is an inflammation of the fluid and lining around the brain. There are different kinds of meningitis, but most of the time it is caused by germs, especially viruses. One such germ that causes meningitis is called tuberculosis (also called TB).
- The purpose of this study is to detect TB meningitis (TBM) earlier and treat TBM better.
- A lumbar puncture (also called a spinal tap) is a test that is performed on all children with meningitis. It is done to evaluate the fluid (spinal fluid) around the brain and spinal cord for germs.
- For this study, we need to take an extra amount of spinal fluid (1 teaspoon) if your child has TB meningitis. Samples will be taken as part of the normal tests that doctors do in all children with meningitis. We will also need to take urine samples from your child. The amount needed is equivalent to 1 teaspoon.
- We also wish to ask you some questions.
- The treatment protocol of your child will not differ from children who do not take part in the study.

## Why have you been invited to take part?

- Your child has been invited to take part in this study because he or she has meningitis.

## What will your responsibilities be?

- We will ask you to answer a few questions. Other than that, there are no responsibilities.

## Will you benefit from taking part in this research?

- Your child might not experience personal benefit from this study. However future patients with TB meningitis will benefit from this study. Information on the results of the study, including new findings, will be available to you at your request.

## Are there in risks involved in your taking part in this research?

- There are no risks involved for you or your child in taking part in this study.

## If you do not agree to take part, what alternatives do you have?

- Whether or not your child takes part in the study this will NOT influence the treatment of your child at all.

## Who will have access to your medical records and obtained samples?

- After collecting samples, these and the other obtained information becomes a secret (anonymized). The samples will be labelled with a study code. This code is a unique code. The child’s identifying information will not be stored for the study. No one will be able to find out the identity of your child anymore.
- The obtained samples will be stored in the NHLS laboratory at the University of Stellenbosch, after which analysis will take place in South Africa. The samples will only be labelled by the study code. So the identity of your child will stay a secret. No one will be able to combine your child’s identifying information with the study results.
- The samples will be destroyed after analysis, as soon as the research project has been completed.
- The information will also remain secret in case the information is used for publication.
- Besides the doctors who treat your child, only the principal investigator will have access to your child’s medical record.

## What will happen in the unlikely event of some form of injury occurring as a direct result of your taking part in this research study?

- There are no risks involved for you or your child in taking part in this study.

Will you be paid to take part in this study and are there any costs involved?

- You or your child will not be paid to take part in the study. There will be no costs involved for you or your child if you take part in the study.

Is there anything else that you should know or do?

- You can contact Dr Solomons xxxxxxxxx 24 hours a day if you have any further queries or encounter any problems.
- You can contact the Health Research Ethics Committee at xxxxxxxxxx if you have any concerns or complaints that have not been adequately addressed by your study doctor. The Health Research Ethics Committee may inspect the study documents at any time.
- You will receive a copy of this information and consent form for your own records.

### Declaration by participant

Inclusion number:

By signing below, I …………………………………..…………. agree to take part in a research study entitled **Improving early diagnosis of tuberculous meningitis in children.**

I declare that:

- I have read or had read to me this information and consent form and it is written in a language with which I am fluent and comfortable.
- I have had a chance to ask questions and all my questions have been adequately answered.
- I understand that taking part in this study is **voluntary** and I have not been pressurised to take part.
- I may choose to leave the study at any time and will not be penalised or prejudiced in any way.
- I may be asked to leave the study before it has finished, if the study doctor or researcher feels it is in my best interests, or if I do not follow the study plan, as agreed to.

Signed at (*place*) ......................…........…………….. on (*date*) …………....……….. 20….

Signature of participant Signature of witness

### Declaration by investigator

I *……………………..* declare that:

- I explained the information in this document to …………………………………..
- I encouraged him/her to ask questions and took adequate time to answer them.
- I am satisfied that he/she adequately understands all aspects of the research, as discussed above
- I did/did not use a interpreter. (*If a interpreter is used then the interpreter must sign the declaration below.*

Signed at (*place*) ......................…........…………….. on (*date*) …………....……….. 20….

Signature of investigator Signature of witness

## Declaration by interpreter

Inclusion number:

I *(name)* ……………………………………………..……… declare that:

- I assisted the investigator (*name*) ………………………………………. to explain the information in this document to (*name of participant*) ……………..…………………………….. using the language medium of Afrikaans/Xhosa.
- We encouraged him/her to ask questions and took adequate time to answer them.
- I conveyed a factually correct version of what was related to me.
- I am satisfied that the participant fully understands the content of this informed consent document and has had all his/her question satisfactorily answered.

Signed at (*place*) ......................…........…………….. on (*date*) …………....……….. 20….

Signature of interpreter Signature of witness

| [](http://www.sun.ac.za/ciguidelines/ciguidelinesafrikaans/Downloads/images/logoalone300.jpg) | STELLENBOSCH UNIVERSITY  **FACULTY OF HEALTH SCIENCES** | 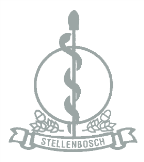 |
| --- | --- | --- |

PARTICIPANT INFORMATION LEAFLET AND ASSENT FORM


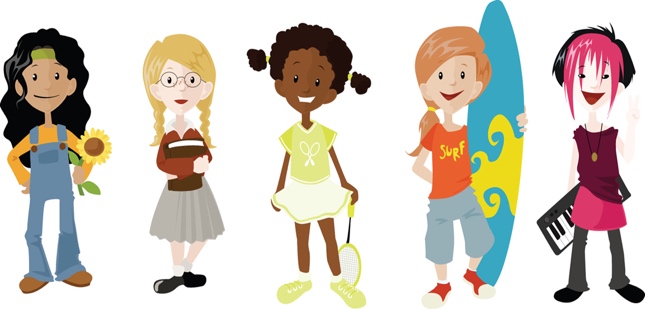

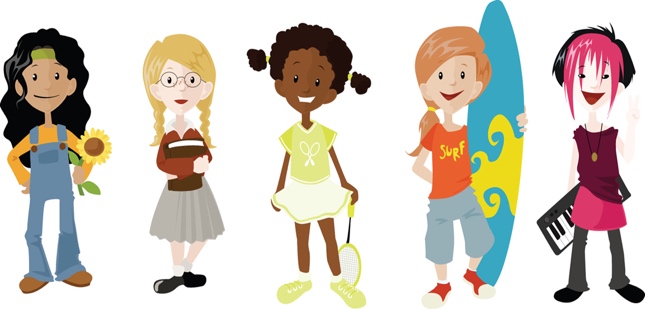


**TITLE OF THE RESEARCH PROJECT:**

Improving early diagnosis of tuberculous meningitis in children

**RESEARCHERS NAME(S): RS Solomons**

**ADDRESS: Department of Paediatrics & Child Health**

**Room 49, Gold Avenue, 3^rd^ Floor, Tygerberg Hospital, Parow Valley, 7503**

**CONTACT NUMBER: xxxxxxxxxx**

## *What is RESEARCH?*

Research is something we do to find new knowledge about the way things (and people) work. We use research projects or studies to help us find out more about disease or illness. Research also helps you to find better ways of helping you when you are sick.

## *What is this research project all about?*

- You are admitted in the Tygerberg Children’s Hospital because of meningitis.
- Meningitis is an inflammation of the fluid and lining around the brain. Most of the time, germs cause meningitis. One such germ that causes meningitis is called tuberculosis (also called TB).
- The purpose of this research project is to pick up TB meningitis earlier in children and therefore to improve how we treat TB meningitis.

## *Why have I been invited to take part in this research project?*

## *You are invited to participate in this research project because you have meningitis.*

**Who is doing the research?**

- Dr Regan Solomons is doing this research project. I am a doctor at Tygerberg Children’s Hospital and I look after children with brain, nerve and muscle problems.

**What will happen to me in this study?**

- To find out if you have meningitis the doctors need to do a lumbar puncture (also called a spinal tap). It is done to look at the fluid (spinal fluid) around the brain and spinal cord for germs.
- By doing a lumbar puncture the doctor puts a needle between the backbones of your lower spine to obtain spinal fluid. The doctor will put some cream on your back so that you will not feel the needle.
- We would like to take an extra amount of fluid from the first lumbar puncture (1 teaspoon). These samples will be taken as part of the normal tests that doctors do in all children with meningitis. No extra punctures will be done.
- We also wish to ask you and your parents some questions.

**Can anything bad happen to me?**

- Nothing bad can happen to you. There will be no extra punctures or other procedures.

**Can anything good happen to me?**

- You will not notice anything good or bad by taking part in this study. But other children with meningitis will benefit from this study in the future. We can better understand how meningitis works, so we can help them better.

**Will anyone know I am in the study?**

- If you decide to take part in this study we keep all the information about you secret. Only your parents and your doctors know that you are taking part in the study.

**Who can I talk to about the study?**

- You can always contact Dr Regan Solomons for questions about the study.
- Tel nr: xxxxxxxxxx

**What if I do not want to do this?**

If you do not want to take part in this study you can stop at any time you want. Also if your parents already gave permission for the study you can stop whenever you want to stop. Your treatment will not be different from children who do take part in the study.

Inclusion number:

Do you understand this research study and are you willing to take part in it?

| YES |  | NO |
| --- | --- | --- |

Has the researcher answered all your questions?

| YES |  | NO |
| --- | --- | --- |

Do you understand that you can pull out of the study at any time?

| YES |  | NO |
| --- | --- | --- |

_________________________ ____________________

Signature of Child Date

**References**

1 Marais S, Thwaites G, Schoeman JF, Török ME, Misra UK, Prasad K, et al. Tuberculous meningitis: a uniform case definition for use in clinical research. Lancet Infect Dis. 2010;10(11):803–12.

2 Mason S, van Furth AM, Mienie LJ, Engelke UF, Wevers RA, Solomons R, et al. A hypothetical astrocyte–microglia lactate shuttle derived from a 1H NMR metabolomics analysis of cerebrospinal fluid from a cohort of South African children with tuberculous meningitis. Metabolomics. 2015;11:822–37.
